# Pathogenic in-Frame Variants in *SCN8A*: Expanding the Genetic Landscape of *SCN8A-*Associated Disease

**DOI:** 10.3389/fphar.2021.748415

**Published:** 2021-11-17

**Authors:** Jennifer C. Wong, Kameryn M. Butler, Lindsey Shapiro, Jacquelyn T. Thelin, Kari A. Mattison, Kathryn B. Garber, Paula C. Goldenberg, Shobana Kubendran, G. Bradley Schaefer, Andrew Escayg

**Affiliations:** ^1^ Department of Human Genetics, Emory University, Atlanta, GA, United States; ^2^ Greenwood Genetic Center, Greenwood, SC, United States; ^3^ Department of Pediatrics and Medical Genetics, Harvard Medical School, Boston, MA, United States; ^4^ Department of Pediatrics, Kansas University School of Medicine-Wichita, Wichita, KS, United States; ^5^ University of Arkansas for Medical Sciences, Little Rock, AR, United States

**Keywords:** SCN8A, sodium channel, epilepsy, seizure, mouse, mutation

## Abstract

Numerous *SCN8A* mutations have been identified, of which, the majority are *de novo* missense variants. Most mutations result in epileptic encephalopathy; however, some are associated with less severe phenotypes. Mouse models generated by knock-in of human missense *SCN8A* mutations exhibit seizures and a range of behavioral abnormalities. To date, there are only a few *Scn8a* mouse models with in-frame deletions or insertions, and notably, none of these mouse lines exhibit increased seizure susceptibility. In the current study, we report the generation and characterization of two *Scn8a* mouse models (ΔIRL/+ and ΔVIR/+) carrying overlapping in-frame deletions within the voltage sensor of domain 4 (DIVS4). Both mouse lines show increased seizure susceptibility and infrequent spontaneous seizures. We also describe two unrelated patients with the same in-frame *SCN8A* deletion in the DIV S5-S6 pore region, highlighting the clinical relevance of this class of mutations.

## Introduction

**FIGURE 1:**
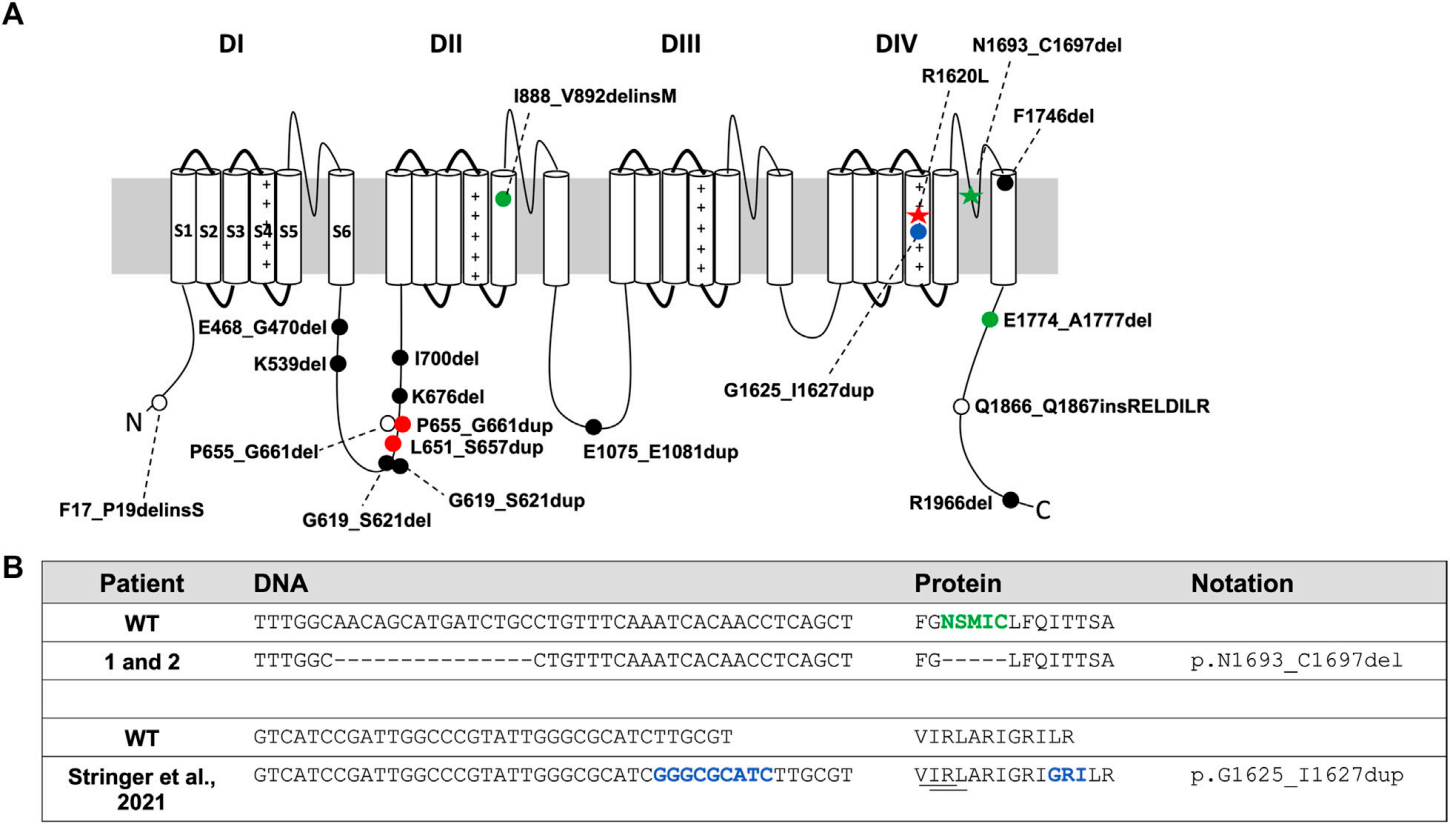
Patient *SCN8A* variants and in-frame variants from gnomAD and ClinVar. **(A)** *SCN8A* channel with variants denoted by: red star, R1620L mutation; green star, in-frame deletion in Patients 1 and 2; filled blue circle, in-frame duplication in proband from Stringer et al., 2021; filled green circle, in-frame variants from Johannesen et al., 2021; open circles, in-frame deletions from ClinVar (July 2021); filled black circle, in-frame deletions and duplications from the gnomAD database (v2.1.1 and v3.1.1; July 2021); filled red circle, in-frame duplication in both gnomAD and ClinVar. **(B)** DNA and protein sequence alignment for Patients 1 and 2 and the proband identified in Stringer et al., 2021. Green indicates amino acids deleted in Patients 1 and 2. Dash (-) indicates a deleted nucleotide or amino acid compared to WT. Blue indicates inserted DNA nucleotides or amino acids. Underline shows close proximity of amino acids deleted in ΔVIR and ΔIRL mouse lines to the variant identified in Stringer et al., 2021.

Voltage-gated sodium channel (VGSC) alpha subunits are comprised of four homologous domains, annotated DI-DIV, and each domain contains six highly conserved transmembrane segments (S1-S6, Figure 1A (Noda et al., 1984; Yu and Catterall, 2003; Catterall, 2012; Catterall, 2000). The S4 segments of VGSCs are enriched with positively charged residues at every third position, and these residues are critical for voltage-dependent gating (Catterall, 2000; Wisedchaisri et al., 2019; Yang et al., 1996; Sokolov et al., 2005). The inactivation gate located between DIII-DIV, and the residues between S5-S6 which form the channel pore, are also highly conserved (Yu and Catterall, 2003; Meisler et al., 2021). Mutations that affect residues in functional domains can impact channel function and alter neuronal excitability (Hodgkin and Huxley, 1952; Catterall, 2000; Yu and Catterall, 2003). Greater variability is observed in the sequence of the intracellular loops between the DI-DII and DII-DIII domains (Figure 1A) (Yu and Catterall, 2003; Meisler et al., 2021).

VGSCs play a critical role in the initiation and propagation of action potentials (Catterall, 2000; Yu and Catterall, 2003; Hu et al., 2009; Catterall, 2012), and mutations in the different members of this gene family are responsible for a wide range of disorders (Brunklaus et al., 2020; Brunklaus and Lal, 2020; Meisler et al., 2021; Wang and Frankel, 2021). Four VGSC alpha subunits are highly expressed in the brain: *SCN1A*, *SCN2A*, *SCN3A* and *SCN8A*. Mutations in each of these genes are responsible for different forms of epilepsy (Brunklaus et al., 2020; Brunklaus and Lal, 2020; Meisler et al., 2021). For example, *SCN1A* mutations are the main cause of genetic epilepsy with febrile seizures plus and Dravet syndrome (Escayg et al., 2000; Lossin, 2009; Escayg and Goldin, 2010). *SCN2A* mutations lead to benign familial neonatal infantile seizures and epileptic encephalopathy (Meisler et al., 2010; Allen et al., 2016; Fukasawa et al., 2015; Saitoh et al., 2015), and *SCN3A* is associated with focal epilepsy (Vanoye et al., 2014; Lamar et al., 2017). The first human *SCN8A* epilepsy-associated mutation (c.5302A > G, p.N1768D) was identified in a proband who presented with spontaneous seizures, behavioral deficits, and sudden unexpected death in epilepsy (SUDEP) (Veeramah et al., 2012). Since this discovery, numerous primarily *de novo* missense *SCN8A* mutations have been identified (Blanchard et al., 2015; Larsen et al., 2015; Wagnon et al., 2016; Butler et al., 2017; Gardella et al., 2018; Johannesen et al., 2018; Brunklaus et al., 2020). Most of the pathogenic *SCN8A* variants are associated with severe epileptic encephalopathy (Blanchard et al., 2015; Larsen et al., 2015; Wagnon et al., 2016; Butler et al., 2017; Gardella et al., 2018; Johannesen et al., 2018; Brunklaus et al., 2020); however, milder phenotypes associated with missense variants have also been reported (Gardella et al., 2016; Rossi et al., 2017; Liu et al., 2019). Truncating mutations (nonsense, frameshift, and splice site alterations) or in-frame deletions/insertions in *SCN8A* are not as frequently observed, and patients with such variants may present with less severe phenotypes (Trudeau et al., 2006; Berghuis et al., 2015). For example, Trudeau et al. identified a patient with a maternally inherited heterozygous truncation in *SCN8A* who presented with cognitive and motor deficits (Trudeau et al., 2006), and Berghuis et al. identified an individual with absence epilepsy, developmental delay, aggression, and attention problems who had a paternally inherited deletion of *SCN8A* exons 2–14 and a maternally inherited missense variant (p.I1583T) (Berghuis et al., 2015).

The first mouse model of *SCN8A* epileptic encephalopathy was generated by knock-in of the *SCN8A* p.N1768D mutation (Wagnon et al., 2015). Heterozygous mice expressing the N1768D mutation recapitulated several phenotypes observed in the original patient, including spontaneous seizures and SUDEP (35). A conditional mouse model with the *SCN8A* R1872W mutation also exhibited spontaneous seizures and early mortality when the mutation was expressed globally in the brain or selectively in excitatory neurons (Bunton-Stasyshyn et al., 2019). Recently, using CRISPR/Cas9 technology, we generated a mouse line expressing the *SCN8A* R1620L mutation (Figure 1A), which was identified in a patient with relatively mild epilepsy and behavioral deficits (Wong et al., 2021a). Mice heterozygous for this mutation exhibit increased seizure susceptibility, spontaneous seizures, impaired learning and memory, social deficits, and altered neuronal excitability (Wong et al., 2021a).

Prior to the development of the mouse lines expressing human *SCN8A* epilepsy mutations, most of the published *Scn8a* mouse models carried loss-of-function missense or truncating *Scn8a* mutations (Martin et al., 2007; Papale et al., 2009; Hawkins et al., 2011; Makinson et al., 2014). Mice heterozygous for loss-of-function *Scn8a* mutations display increased resistance to induced seizures (Martin et al., 2007; Hawkins et al., 2011; Makinson et al., 2014), and depending on the genetic background, some lines also exhibit spike-wave discharges (Papale et al., 2009). There are currently only a few *Scn8a* mouse models with in-frame deletions or insertions (Jones et al., 2016; Inglis et al., 2020), and notably, none of these mouse lines show increased seizure susceptibility. Jones et al., 2016 reported an in-frame deletion (p.I1750del) in the DIVS6 domain, and homozygous mutants with this mutation exhibited motor impairments and early mortality (Jones et al., 2016). We previously generated two *Scn8a* mouse lines, one with an in-frame deletion (p.R848_F850del, Δ9) and the other with an in-frame insertion (p.R848_V849insD, ∇3) in the DIIS4 (Inglis et al., 2020). Heterozygous mutants from both of these lines exhibit increased seizure resistance (Inglis et al., 2020). In the current study, we report the generation and characterization of two novel *Scn8a* mouse models with overlapping in-frame deletions in the DIVS4 that exhibit increased seizure susceptibility and spontaneous seizures. We also describe two unrelated patients with the same in-frame *SCN8A* deletion in the DIV S5-S6 pore region*,* highlighting the clinical relevance of in-frame *SCN8A* mutations.

## Materials and Methods

### Animals

**FIGURE 2:**
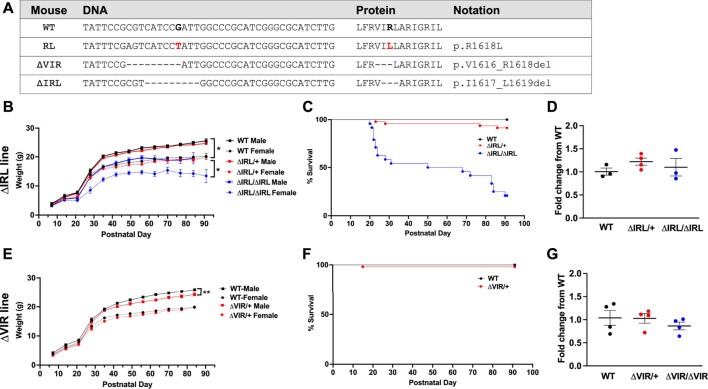
Generation and characterization of the ΔIRL and ΔVIR mouse lines. **(A)** DNA and protein sequence alignment. WT, wild-type mouse sequence, RL, the human R1620L mutation (red) in the RL mouse line, ΔVIR, the 3 amino acid deletion in the ΔVIR mouse line, ΔIRL, the 3 amino acid deletion in the ΔIRL mouse line. Dash (-) indicates a deleted nucleotide or amino acid when compared to the WT sequence. **(B)** Homozygous ΔIRL/ΔIRL mutants weigh significantly less than same-sex heterozygous ΔIRL/+ mutants and WT littermates. Kruskal-Wallis test followed by Dunn’s multiple comparisons. **(C)** Approximately 25% of ΔIRL/ΔIRL mutants and 90% of ΔIRL/+ mutants survive to postnatal day 90 (P90). Log-rank Mantel Cox test. **(B and C)** WT male *N* = 10; WT female *N* = 11; ΔIRL/+ male *N* = 29; ΔIRL/+ female *N* = 17, ΔIRL/ΔIRL male *N* = 13; ΔIRL/ΔIRL female *N* = 11. **(D)** *Scn8a* mRNA expression levels were comparable for the three genotypes in the ΔIRL mouse line. Kruskal-Wallis test. *N* = 3–4/genotype. **(E)** Heterozygous ΔVIR/+ male mutants weigh significantly less than same-sex WT littermates. Female ΔVIR/+ mutants and WT littermates had comparable weights. Paired Student's *t* test. **(F)** Approximately 95% of ΔVIR/+ mutants survive to P90. Log-rank Mantel Cox test. **(E and F)** WT male *N* = 27; WT female *N* = 22; ΔVIR/+ male *N* = 18; ΔVIR/+ female *N* = 36. **(G)** *Scn8a* mRNA expression levels were comparable for the three genotypes in the ΔVIR mouse line. Kruskal-Wallis test. *N* = 4/genotype. *p ≤ 0.05, **p ≤ 0.01.

Using CRISPR/Cas9, we knocked in the human *SCN8A* p.R1620L mutation into the mouse *Scn8a* gene on the C57BL/6J background (corresponding to R1618L in the mouse, Figure 2A (Wong et al., 2021a). As a result of nonhomologous end joining during this process, we generated two additional *Scn8a* mouse lines (*Scn8a* ^9Δ_IRL^ and *Scn8a* ^9Δ_VIR^) with overlapping 9 base pair in-frame deletions which include removal of the positively charged R1618 residue (Figure 2A). Heterozygous mutants from the *Scn8a* ^9Δ_IRL^ (ΔIRL/+) and *Scn8a* ^9Δ_VIR^ (ΔVIR/+) lines were backcrossed to C57BL/6J mice (Strain: 000664, Jackson Laboratories) for four generations. We performed Sanger sequencing on mutant mice from each line to confirm that the in-frame deletions were the only changes in *Scn8a* exon 26 and that the conserved exon 26 of the other brain sodium channels, *Scn1a, Scn2a, and Scn3a,* were unaltered. The 9 bp deletions were the only alterations observed in *Scn8a* exon 26 and no off-target CRISPR editing was observed in exon 26 of the other sodium channels. Male and female ΔIRL/+ and ΔVIR/+ mutants and respective wild-type (WT) littermates at the N4 generation were used for all experiments. To examine weight gain and survival of ΔIRL mice, male and female heterozygous mutants were bred to generate homozygous ΔIRL/ΔIRL mutants, heterozygous ΔIRL/+ mutants, and WT littermates. We tried to similarly breed the ΔVIR mouse line, however an insufficient number of litters were generated. Therefore, for the ΔVIR mouse line, heterozygous male mutants were bred with C57BL/6J females to generate heterozygous ΔVIR/+ mutants and WT littermates.

To generate biallelic mice expressing the R1620L and ΔVIR mutations, male ΔVIR/+ mutants were crossed with female RL/+ mutants to generate the following genotypes: ΔVIR/+, RL/+, ΔVIR/RL, and WT. This breeding scheme allowed us to examine littermates expressing the R1620L and ΔVIR mutations. Survival and weights were recorded. Mice were housed on a 12 h light/dark cycle with food and water *ad libitum*. All experiments were performed in accordance with the guidelines of the Institutional Animal Care and Use Committee of Emory University.

### Genotyping

DNA was isolated from tail biopsies. Mice with the 9Δ_IRL or 9Δ_VIR allele were identified by PCR amplification using primer R1620L_R1: TAC​GCG​AAG​TTG​GAC​ATC​CC and forward primers that span the respective 9 bp deletions: Scn8a_9del_IRL_F1: CCC​TAT​TCC​GCG​TGG​CCC and Scn8a_9del_VIR_F2: TCT​CCC​CGA​CCC​TAT​TCC​GAT​T. The 9Δ_IRL and 9Δ_VIR alleles generated 175 bp and 184 bp PCR products, respectively. The WT allele was identified using the primers R1620L_R1 and Scn8a_WT_F: CTA​TTC​CGC​GTC​ATC​CGA​TTG​G, which generated a 183 bp product. The RL/+ mutants were genotyped as previously described (Wong et al., 2021a).

### qRT-PCR

Whole brains were extracted from P20-P21 WT, heterozygous, and homozygous mutants of both sexes from each mouse line. RNA extraction and cDNA synthesis were performed as previously described (Lamar et al., 2017). The following *Scn8a* primer pair was used: Scn8a_F: AGA​TTT​AGC​GCC​ACT​CCT​GC and Scn8a_R: GGA​CCA​TTC​GGG​AGG​GTT​AC. Analyses were conducted in technical triplicates using the Real-Time PCR Detection System and SYBR Green (BioRad). Expression levels were normalized to beta-actin (F: CAG​CTT​CTT​TGC​AGC​TCC​TT and R: ACG​ATG​GAG​GGG​AAT​ACA​GC). Relative expression between genotypes was compared using the ΔΔct method.

### 6 Hz Seizure Induction

Seizures were induced using the 6 Hz seizure induction paradigm as previously described (Barton et al., 2001; Devinsky et al., 2016; Wong et al., 2016; Lamar et al., 2017; Shapiro et al., 2019; Wong et al., 2019; Wong et al., 2021b). Briefly, ΔIRL/+ and ΔVIR/+ mutants and their WT littermates of both sexes (2 months old) were subjected to corneal stimulation (6 Hz, 0.2 ms pulse width, 3 s) using a constant current device (ECT unit, 57800; Ugo Basile, Comerio, Italy). The ΔIRL and ΔVIR mouse lines were tested at 14 and 16 mA, respectively. Behavioral seizures were scored using a modified Racine scale (RS): RS0, no abnormal behavior; RS1, immobile ≥3 s; RS2, forelimb clonus, head bobbing, paw waving; and RS3, rearing and falling. A greater RS value indicates a more severe behavioral seizure.

### Flurothyl Seizure Induction

ΔIRL/+, ΔVIR/+, RL/+, and their WT littermates (2 months old) of both sexes were individually placed into a clear acrylic chamber and flurothyl (2,2,2-trifluroethylether, Sigma-Aldrich) was introduced into the chamber at 20 μL/min as previously described (Prichahd et al., 1969; Wong et al., 2016; Shapiro et al., 2019). The latencies to the first myoclonic jerk (MJ) and generalized tonic-clonic seizure (GTCS) were recorded for each mouse.

### EEG Surgery and Analyses

Cortical electrodes were implanted into adult (2–4 months old) male ΔIRL/+ and male ΔVIR/+ mutants as previously described (Lamar et al., 2017; Wong et al., 2018; Inglis et al., 2020; Shapiro et al., 2019). Four cortical screw electrodes were implanted into the skull at the following coordinates relative to bregma: anterior-posterior(AP) +2.0 mm and medial-lateral(ML) +1.2 mm; AP −1.5 mm and ML +1.2 mm; AP +0.5 mm and ML −2.2 mm; and AP −3.5 mm and ML −2.2 mm. Two fine-wire electrodes were implanted into the neck muscles for EMG recordings. Each mouse was allowed 1 week to recover from the surgical procedure. Stellate Harmonie rodent software was used to obtain and analyze EEG recordings. Seizures were manually identified and characterized by high frequency and amplitude EEG signals that were at least 3 s in duration and twice the background. Simultaneous video recordings were used to confirm behavioral seizures.

### Next-Generation Sequencing of Patients

Two unrelated patients (Patients 1 and 2) had targeted gene panel sequencing performed by GeneDx. Clinical information was provided by the corresponding clinician in each case. The corresponding author can be contacted for additional information on the patients described in this manuscript. This study was approved by the Institutional Review Board of Emory University.

### Databases

Three databases were used for the identification of in-frame *SCN8A* variants: 1) the Genome Aggregation database (gnomAD, v2.1.1 and v3.1.1; https://gnomad.broadinstitute.org) (Karczewski et al., 2020), 2) ClinVar (https://www.ncbi.nlm.nih.gov/clinvar/) (Landrum et al., 2018), and 3) the Human Gene Mutation database (HGMD, v2021.2; http://www.hgmd.cf.ac.uk/ac/index.php). The gnomAD database is a compilation of exome and genome sequencing data from a variety of large-scale sequencing projects, and efforts have been made to remove individuals affected by severe pediatric disease. There are currently two versions of the gnomAD database: v2.1.1 contains both exome and whole genome sequences (141,456 samples, GRCh37 build), while v3.1.1 contains only whole genome sequences (76,156 samples, GRCh38 build). Approximately 20,000 genomes overlap between v2.1.1 and v3.1.1. The ClinVar database is a freely available, non-curated, public repository of human genetic variants. Interpretation of the significance of these variants to disease is provided by the submitter and does not require experimental validation. Submitters include clinical testing laboratories, research laboratories, expert panels, and other groups that must be approved by the National Center for Biotechnology Information (NCBI). The HGMD database is a curated collection of published genetic variants that are associated with human disease.

### Statistical Analyses

Data are presented as mean ± SEM with *p* ≤ 0.05 considered as statistically significant. All statistical analyses were performed with Prism 9.0 (GraphPad Software, San Diego, CA). A Chi-square test was used to compare the observed number of offspring with the expected ratio. A log-rank Mantel Cox test was used to compare survival curves. A Kruskal-Wallis test followed by Dunn’s multiple comparisons test was used to compare weights within the ΔIRL mouse line. A paired Student’s *t* test was used to compare weights between ΔVIR/+ mutants and same-sex WT littermates. A Kruskal-Wallis test was used to compare *Scn8a* expression levels between genotypes. A Mann-Whitney test was used to compare Racine scores following 6 Hz-induced seizures between mutants and WT littermates. An unpaired Student’s *t* test was used to compare the latency to the first MJ and GTCS between mutants and WT littermates from the ΔVIR and ΔIRL mouse lines. A Kruskal-Wallis test followed by Dunn’s multiple comparisons test was used to compare the latency to the first MJ and GTCS between the mutants and WT littermates from the ΔVIR x RL matings.

## Results

### Survival

Male and female ΔIRL/+ mutants were crossed to generate homozygous ΔIRL/ΔIRL, heterozygous ΔIRL/+, and WT littermates. From a total of 9 litters, we observed 21 WT (10 males, 11 females), 46 ΔIRL/+ (29 males, 17 females), and 24 ΔIRL/ΔIRL (13 males, 11 females) offspring, which is consistent with the predicted 1:2:1 Mendelian ratio (*p* = 0.21). ΔIRL/ΔIRL mutants weighed significantly less than sex-matched ΔIRL/+ mutants and WT littermates (*p* ≤ 0.05, Figure 2B), and ΔIRL/+ and ΔIRL/ΔIRL mutants exhibited approximately 90 and 25% survival at postnatal day 90 (P90), respectively (Figure 2C). Heterozygous ΔVIR/+ female mutants did not breed efficiently, and we were unable to obtain enough offspring to generate a survival curve. As an alternative, we mated male ΔVIR/+ mutants with C57BL/6J females in order to examine the survival of heterozygous ΔVIR/+ mutants. Male ΔVIR/+ mutants weighed significantly less than sex-matched WT littermates (*p* ≤ 0.01, Figure 2E), and one ΔVIR/+ male did not survive to P90 (Figure 2F). In contrast, none of the ΔVIR/+ females died, and their body weights were comparable to same-sex WT littermates (Figure 2E). From 16 litters, we observed 54 ΔVIR/+ mutants (18 males, 36 females) and 49 WT littermates (27 males, 22 females), which is consistent with the expected 1:1 ratio (*p* = 0.78).

### mRNA Expression

Quantitative real-time RT-PCR analysis was performed on whole brain samples from P20-P22 mice of each genotype from the ΔIRL and ΔVIR mouse lines. We observed similar levels of *Scn8a* expression within and between each mouse line (Figures 2D–G).

### Heterozygous ΔIRL/+ and ΔVIR/+ Mutants Exhibit Increased Seizure Susceptibility

**FIGURE 3:**
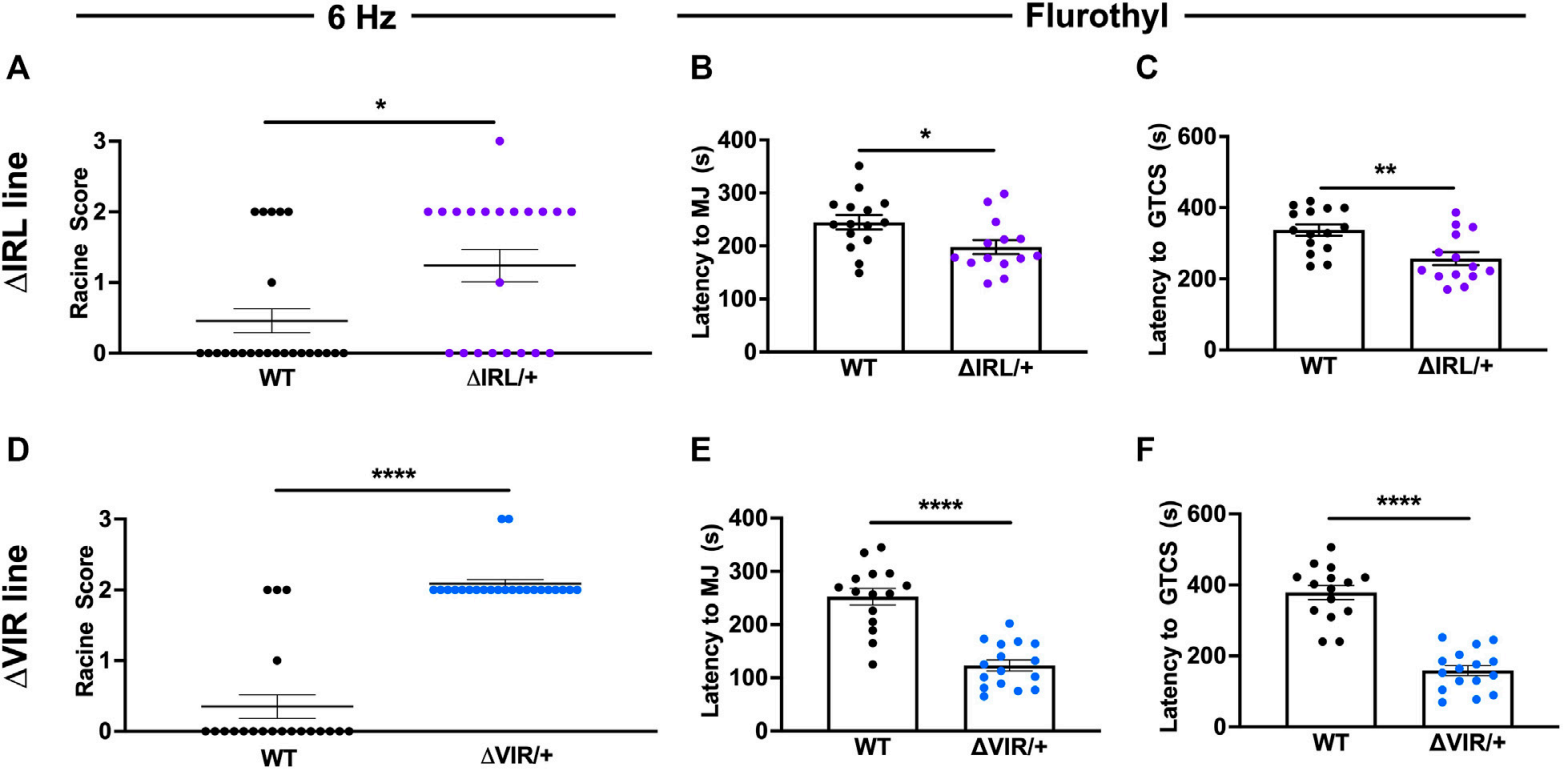
**Δ**IRL/+ and ΔVIR/+ mutants exhibit increased seizure susceptibility. **(A)** ΔIRL/+ mutants are significantly more susceptible to 6 Hz-induced seizures compared to WT littermates. Mann-Whitney test. WT *N* = 25; ΔIRL/+ *N* = 21. **(B,C)** ΔIRL/+ mutants exhibit a lower average latency to the first MJ (**B**) and GTCS **(C)** compared to WT littermates. Unpaired student’s *t* test. WT *N* = 15; ΔIRL/+ *N* = 14. **(D)** ΔVIR/+ mutants are more susceptible to 6 Hz-induced seizures compared to WT littermates. Mann-Whitney test. WT *N* = 20; ΔVIR/+: *N* = 23. **(E,F)** ΔVIR/+ mutants exhibit lower average latencies to the first MJ **(E)** and GTCS **(F)** compared to WT littermates. Unpaired Student's *t* test. WT *N* = 15; ΔVIR/+ *N* = 16. *p ≤ 0.05, **p ≤ 0.01, ****p ≤ 0.0001.

Susceptibility to 6 Hz- and flurothyl-induced seizures were compared between heterozygous mutants (ΔIRL/+ or ΔVIR/+) and WT littermates from each line. We did not observe any statistically significant differences in susceptibility to 6 Hz- or flurothyl-induced seizures between the sexes; therefore, data from both sexes were combined for analysis. Both ΔIRL/+ and ΔVIR/+ mutants were significantly more susceptible to 6 Hz-induced seizures when compared to their respective WT littermates (Figures 3A,D). For the ΔIRL/+ mutants, 12/20 mice seized (RS = Racine Score; 8RS0, 1 RS1, 11 RS2, 1 RS3) when compared to 6/25 WT littermates (19 RS0, 1 RS1, 5 RS2). Similarly, all of the ΔVIR/+ mutants exhibited a 6 Hz seizure (21 RS2, 2 RS3) whereas most of the WT littermates did not seize (16 RS0, 1 RS1, 3 RS2). When tested with flurothyl, ΔIRL/+ and ΔVIR/+ mutant mice exhibited significantly shorter latencies to the first myoclonic jerk (Figures 3B,E) and the first GTCS (Figures 3C,F) when compared to their WT littermates. Although we cannot directly compare the two mouse lines, the average latencies to the first MJ and GTCS in the ΔVIR/+ mutants were approximately 40% shorter than the corresponding measurements from the ΔIRL/+ mutants, suggesting that the ΔVIR/+ mutants may be more severely affected.

### Heterozygous ΔIRL/+ and ΔVIR/+ Mutants Exhibit Spontaneous Seizures

**FIGURE 4:**
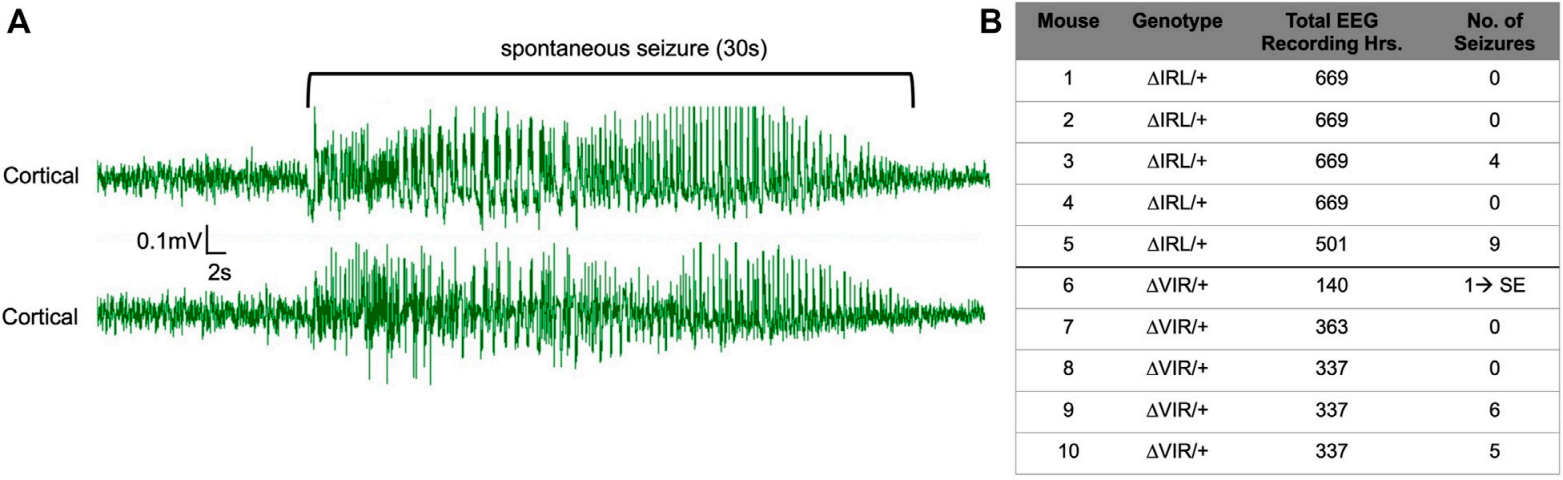
ΔIRL/+ and ΔVIR/+ mutants exhibit spontaneous seizures. **(A)** Representative cortical EEG traces from a ΔVIR/+ mutant before, during, and after a spontaneous seizure. **(B)** Number of spontaneous seizures observed in 5 male ΔIRL/+ and 5 male ΔVIR/+ mutants. SE denotes status epilepticus.

Continuous EEG recordings were obtained from five male ΔIRL/+ and five male ΔVIR/+ mutants. 2/5 ΔIRL/+ mutants and 3/5 ΔVIR/+ mutants exhibited spontaneous seizures. Figure 4A provides an example of a spontaneous seizure observed in a ΔVIR/+ mutant. Spontaneous seizures were between 17–60 s in duration with the exception of one ΔVIR/+ mutant (Mouse #6, Figure 4B) that had a 4 min-long seizure, followed by 2 min of recovery, entry into status epilepticus for approximately 4 h, and ultimately, death. Spontaneous seizure frequency was infrequent, with an average seizure frequency of less than 1 seizure/day in the mice that exhibited seizures (Figure 4B). Electrographic seizures were accompanied by rearing, paw waving, loss of posture, and in some instances, wild running and bouncing.

### Biallelic Mutants Exhibit Premature Mortality and Increased Seizure Susceptibility

**FIGURE 5:**
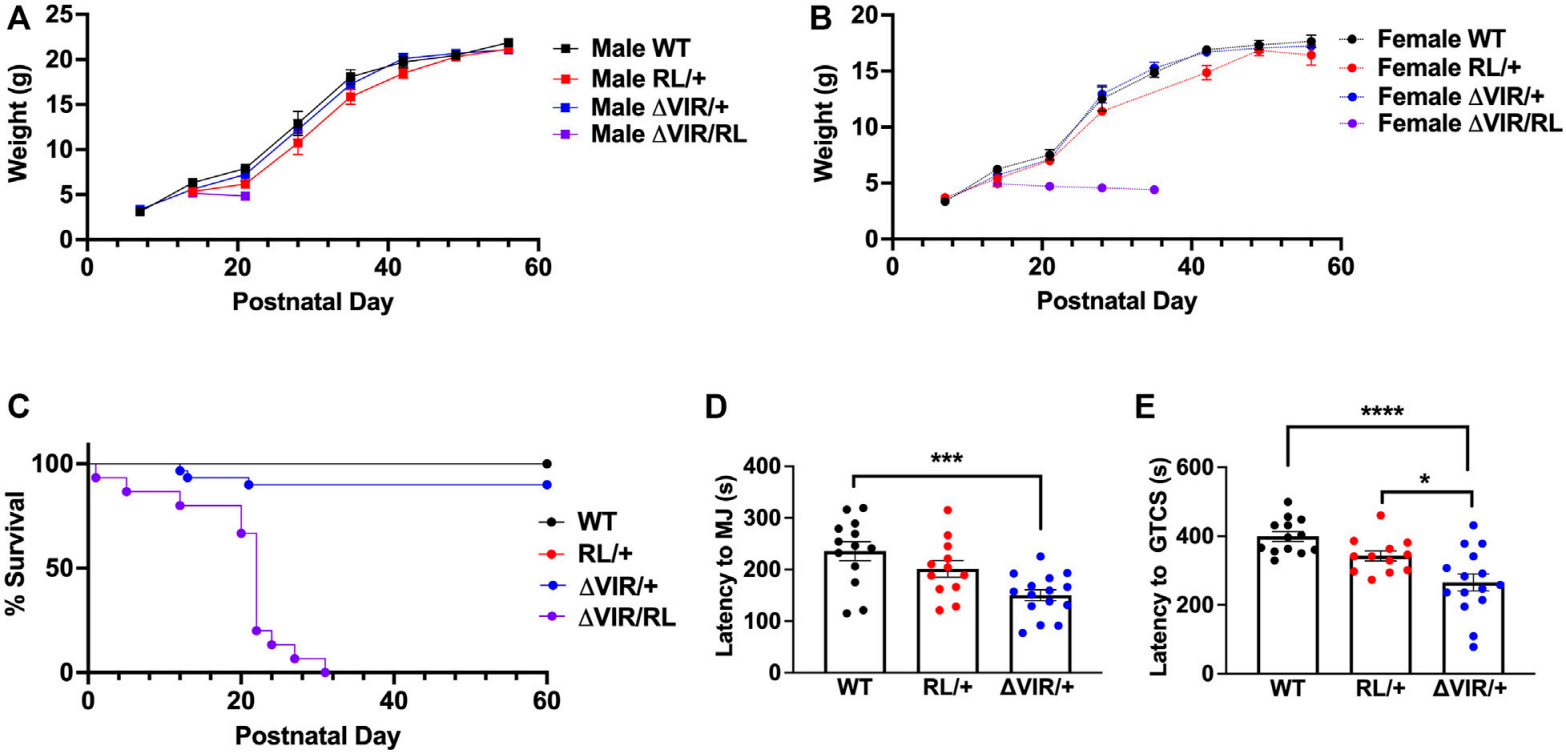
Generation and characterization of ΔVIR/RL biallelic heterozygous mice. **(A,B)** ΔVIR/RL mutants weighed less than same-sex littermates from P12. **(C)** Male and female ΔVIR/RL mutants exhibited a maximum lifespan of 22 and 31 days, respectively. Only 3/22 ΔVIR/+ mutants died prematurely. Log-rank Mantel Cox test. **(A-C)** WT male *N* = 12; WT female *N* = 10; RL/+ male *N* = 12; RL/+ female *N* = 3; ΔVIR/+ male *N* = 16; ΔVIR/+ female *N* = 14; ΔVIR/RL male *N* = 7; ΔVIR/RL female *N* = 8. **(D)** Compared to WT littermates, ΔVIR/+ mutants exhibited a significantly lower average latency to the first MJ. **(E)** ΔVIR/+ mutants exhibit a significantly lower average latency to the first GTCS compared to RL/+ mutants and WT littermates. **(D,E)** Kruskal-Wallis test followed by Dunn’s multiple comparisons. WT *N* = 13; RL/+ *N* = 12; ΔVIR/+ *N* = 15. *p ≤ 0.05, ***p ≤ 0.001, ****p ≤ 0.0001.

Based on our previous characterization of the RL mutants (Wong et al., 2021a), we know that homozygous RL mutants gain weight normally until postnatal day 15 (P15) and have a maximum lifespan of 22 days. Thus, to examine the relative severity of the ΔVIR mutation compared to the pathogenic human *SCN8A* p.R1620L mutation (Wong et al., 2021a), we crossed heterozygous male ΔVIR/+ mutants with heterozygous female RL/+ mutants to generate WT, ΔVIR/+, RL/+, and biallelic (ΔVIR/RL) littermates. Beginning at P12, ΔVIR/RL mutants of both sexes were smaller than their same-sex littermates (Figures 5A,B). Male and female ΔVIR/RL mutants exhibited premature death, and maximum lifespans of 22 and 31 days were observed, respectively (Figure 5C).

We observed no sex differences in the latency to the first MJ or GTCS following flurothyl exposure; therefore, we combined the data from male and female mice of the same genotype. Although not statistically significant, the RL/+ mutants exhibited a lower average latency to the first MJ and GTCS compared to WT littermates (Figures 5D,E). Average latency to the first MJ was significantly lower in ΔVIR/+ mutants compared to WT littermates (Figure 5D), and the average latency to the first GTCS was significantly lower in ΔVIR/+ mutants compared to RL/+ mutants and WT littermates (Figure 5E), suggesting greater severity of the ΔVIR mutation. Due to the premature death of the ΔVIR/RL mutants, we were unable to evaluate their susceptibility to flurothyl-induced seizures.

### Patients With in-Frame *SCN8A* Variants

#### Patients 1 and 2: c.5077_5091del, p.Asn1693_Cys1697del

Gene panel testing of two unrelated patients (Patient 1 and Patient 2) identified the same heterozygous in-frame deletion variant, *SCN8A* c.5077_5091del (p.N1693_C1697del) (Figure 1B), located in the DIV S5-S6 pore region (Figure 1A, green star). The c.5077_5091del variant is currently classified as a “variant of uncertain clinical significance” (Richards et al., 2015). Patient 1 presented with autism, developmental delay, dysmorphic facial features, but no seizures. The deletion was determined to be inherited from the unaffected mother. Patient 2 presented with autism, encephalopathy with developmental delay, tremors, facial asymmetry, low muscle tone, skeletal abnormalities, and femoral torsion, but no history of seizures. The deletion was determined to be *de novo* for Patient 2*.* Patient 2 also had another *de novo* heterozygous variant in *ANK3* c.3821C > A (p.S1274Y), which was classified as a “variant of uncertain clinical significance.”

## Discussion

In the current manuscript, we describe the generation and characterization of two *Scn8a* mouse lines (ΔIRL and ΔVIR) with overlapping, in-frame deletions. Heterozygous ΔIRL/+ and ΔVIR/+ mutants exhibit increased seizure susceptibility and spontaneous seizures, demonstrating the potential for this class of genetic variation to contribute to the clinical burden associated with *SCN8A* dysfunction. Consistent with this, we also report the identification of an in-frame *SCN8A* variant in two unrelated patients with neurodevelopmental phenotypes, but no seizures.

**FIGURE 6:**
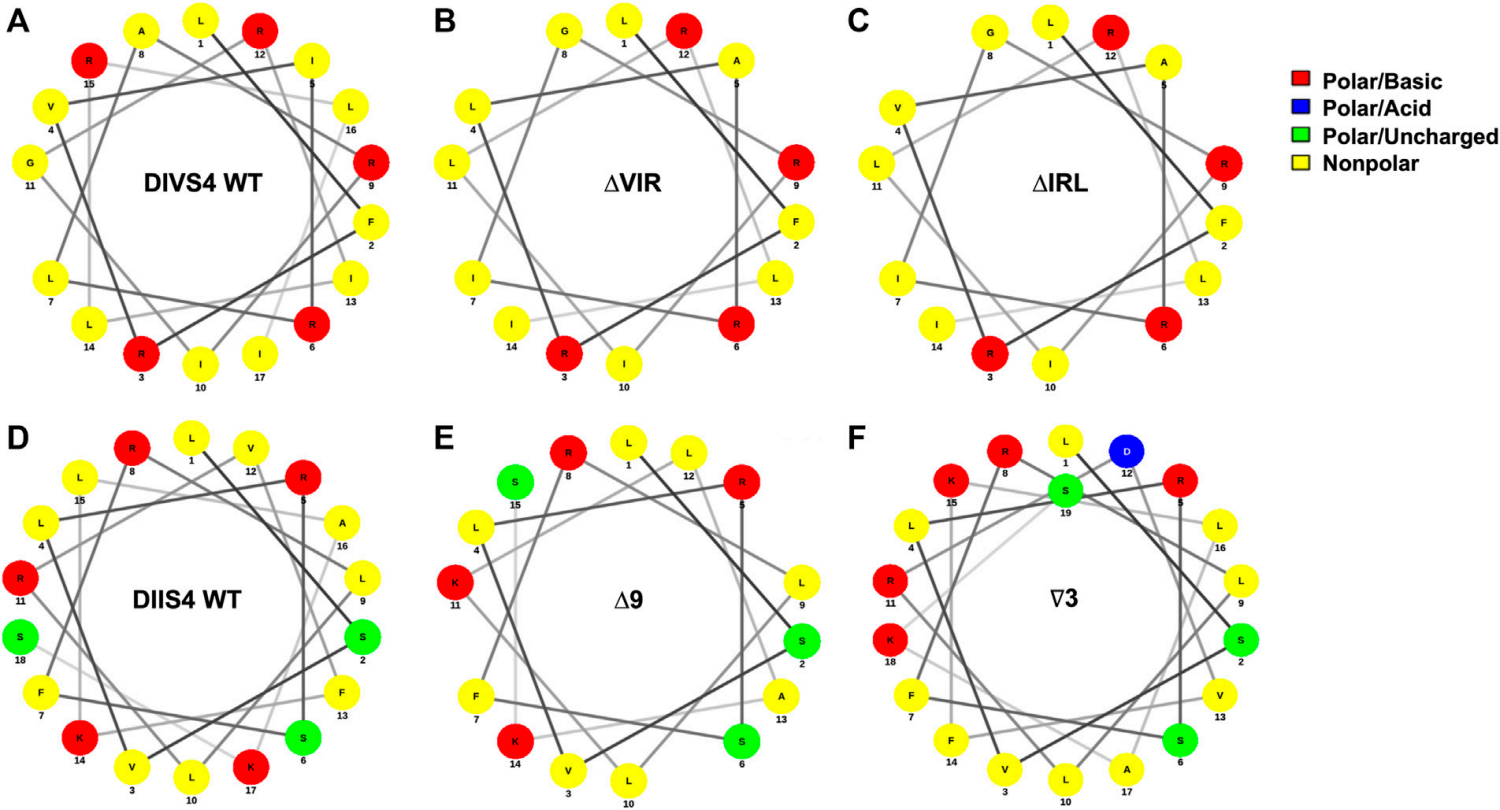
*In silico* analysis of in-frame deletions in the DIVS4 and DIIS4 transmembrane segments. **(A–C)** Alpha helix wheel diagrams of the WT DIVS4 transmembrane segment **(A)**, the ΔVIR mutation **(B)**, and the ΔIRL mutation **(C)**. **(D–F)** Alpha helix wheel diagrams of the WT DIIS4 transmembrane segment **(D)**, the Δ9 mutation **(E)**, and the ∇3 mutation **(F)** from Inglis et al., 2020. The alpha helix wheel diagrams were generated using NetWheels (http://lbqp.unb.br/NetWheels/).

We previously observed increased resistance to induced seizures in heterozygous *Scn8a* ^Δ9/+^ and *Scn8a* ^∇3/+^ mutant mice, expressing an in-frame 9 bp deletion (Δ9) and 3 bp insertion (∇3) in the DIIS4, respectively (Inglis et al., 2020). EEG analyses of the *Scn8a* ^Δ9/+^ mutants revealed normal electrographic activity and no spontaneous seizures (Inglis et al., 2020). In contrast, ΔIRL/+ and ΔVIR/+ mutants, harboring overlapping 9 bp deletions within the DIVS4, exhibited increased seizure susceptibility and spontaneous seizures. We performed *in silico* analyses of the DIIS4 and DIVS4 transmembrane domains (Figure 6) and found that the Δ9 mutation in the DIIS4 causes a loss of a positive charge and shift of polar charges. The ∇3 mutation in DIIS4 causes a shift of the polar charges to one side of the helix. In contrast, the VIR and IRL deletions in DIVS4 do not significantly alter the structure and distribution of charges in the alpha helix. Differences between the phenotypes of mice expressing the Δ9 and ∇3 mutations versus the ΔVIR and ΔIRL mutations may also be due, in part, to differences in the functional properties of the domains, with DI-DIII primarily for activation (Kontis et al., 1997; Yu and Catterall, 2003) and DIV primarily involved in inactivation (Kontis and Goldin, 1997; Yu and Catterall, 2003). These contrasting observations also highlight that the phenotypic consequences of in-frame deletions or insertions may likely be difficult to predict. Additional studies are warranted to characterize the biophysical impact of the ΔIRL and ΔVIR alleles relative to other *SCN8A* missense and in-frame variants.

To date, most identified human *SCN8A*-epilepsy associated mutations have been *de novo* amino acid substitutions, many of which are predicted or shown to have gain-of-function properties (Veeramah et al., 2012; Estacion et al., 2014; Liu et al., 2019; Heyne et al., 2020). A small number of truncating and frameshift mutations have also been described and appear to contribute to neurodevelopmental phenotypes such as autism and intellectual disability (Brunklaus et al., 2020; Brunklaus and Lal, 2020). Of direct clinical relevance, we report two unrelated individuals with the same in-frame *SCN8A* variant. Patient 1 and Patient 2 both carry the same in-frame deletion (p.N1693_C1697del) which removes five amino acids (Figure 1B) from the DIV S5-S6 pore region (Figure 1A). Previous genotype-phenotype correlations found that variants in the pore region were more likely to be associated with loss-of-function rather than gain-of-function effects and epilepsy (Holland et al., 2018; Brunklaus et al., 2020). Consistent with this, Patients 1 and 2 exhibit developmental delay and autism without seizures. Although this variant was not observed in the gnomAD database, additional functional studies will be necessary to resolve its clinical significance. This is particularly relevant since the p.N1693_C1697del variant occurred *de novo* in Patient 2, but in Patient 1, was inherited from an unaffected parent. Patient 2 was also found to harbor a *de novo* substitution (p.S1274Y) in *ANK3,* which is important for connecting integral proteins with the spectrin-actin cytoskeleton (Kordeli and Bennett, 1991). *ANK3* is associated with both autosomal dominant and recessive neurodevelopmental disorders, including intellectual disability and autism spectrum disorder (Bi et al., 2012; Iqbal et al., 2013; Kloth et al., 2017; Hu et al., 2019). *In silico* algorithms suggest a possible deleterious effect of the p.S1274Y variant (e.g., CADD = 26.3); however, this variant is observed twice in the gnomAD database (v3.1.1), and there are no pathogenic variants in the HGMD database that are in close proximity to this variant. Interestingly, *ANK3* has been shown to associate with VGSCs *via* a binding site on the DII-DIII intracellular loop (Jenkins and Bennett, 2001; Lemaillet et al., 2003; Gasser et al., 2012), raising the possibility that it may act as a genetic modifier of *SCN8A.*

Stringer and others recently described a patient with epileptic encephalopathy who harbors a *de novo* heterozygous in-frame duplication in *SCN8A* (p.G1625_I1627dup) and an inherited heterozygous missense variant in *CACNA1H* (p.G318S) (Stringer et al., 2021). The p.G1625_I1627dup variant (Figure 1A, blue circle), located in the DIVS4 transmembrane domain, is proximal to several pathogenic variants, including p.R1620L (Rossi et al., 2017; Liu et al., 2019) and p.A1622D (Liu et al., 2019). This duplication results in the insertion of three amino acids, including an additional positively charged arginine residue (Figure 1B). Interestingly, while this duplication does not overlap with the amino acids altered in the ΔIRL and ΔVIR mice, it is immediately adjacent to them (Figure 1B). The p.G1625_I1627dup variant resulted in a hyperpolarizing shift of the voltage-dependence of activation of Na_v_1.6 but had no effect on sodium current density or gating mechanisms (Stringer et al., 2021). This observation is consistent with a gain-of-function effect on the Na_v_1.6 channel; however, Stringer et al. also demonstrated that the c.952G > A, p.G318S variant in *CACNA1H* was associated with loss-of-function effects. Further work will be required to resolve the relative contribution of each variant to the clinical presentation.

Johannesen et al. also recently reported two “likely pathogenic” in-frame *SCN8A* variants in a large cohort of Danish patients with *SCN8A* mutations (Johannesen et al., 2021). The maternally inherited p.I888_V892delinsM variant was identified in one patient with moderate intellectual disability without epilepsy (Johannesen et al., 2021). Another patient had a maternally inherited variant (E1774_A1777del) and presented with several types of seizures, including febrile, myoclonic, and atonic seizures, but normal intellect (Johannesen et al., 2021). It is unclear whether the mothers of these patients displayed similar clinical features.

There are at least six in-frame variants in the ClinVar database (Table 1; Figure 1A), including the p.N1693_C1697del variant observed in Patients 1 and 2. One ClinVar variant (p.Q1866_Q1867insRELDILR) introduces seven amino acids in the C-terminus (Figure 1A) and is classified as “likely pathogenic”. This variant is not observed in the gnomAD database (v3.1.1), and it is adjacent to several reported variants associated with epileptic encephalopathy, including p.L1865P (Trump et al., 2016), E1870D (Boerma et al., 2016), and R1872 which is one of the most frequently mutated SCN8A residues (Ohba et al., 2014; Larsen et al., 2015; Wagnon et al., 2016). Examination of the population gnomAD database identified 11 in-frame variants in *SCN8A* (Table 2; Figure 1A). Whether these variants alter the biophysical properties of the channel is currently unknown; however, most of these variants are located in the intracellular DI-DII and DII-DIII linkers (Figure 1A), which are generally more tolerant of variation (Yu and Catterall, 2003; Meisler et al., 2021). In contrast, pathogenic *SCN8A* variants are typically located in the more conserved parts of the channel, such as the transmembrane segments, inactivation gate, and pore (Wagnon and Meisler, 2015; Brunklaus et al., 2020).

**TABLE 1:** In-frame *SCN8A* variants observed in the ClinVar database.

Transcript change	Protein change	Location	gnomAD v2.1.1 allele count	gnomAD v3.1.1 allele count	Classification	Clinical info
c.50_55delTCACC	p.F17_P19delinsS	N-terminus	0	0	VUS	EIEE
c.1952_1972dupTCATCGGCGGCCCCGGCTCCC	p.L651_S657dup	DI-DII Linker	0	1	VUS	EIEE
c.1962_1982dupCCCCGGCTCCCACATCGGCGG	p.P655_G661dup	DI-DII Linker	1	0	VUS	EIEE
c.1962_1982delCCCCGGCTCCCACATCGGCGG	p.P655_G661del	DI-DII Linker	0	0	VUS	NS
c.5077_5091delAACAGCATGATCTGC	p.N1693_C1697del^a^	DIV pore region	0	0	VUS	NS
c.5579_5599dupGGGAGTTGGACATCCTGCGGC	p.Q1866_Q1867insRELDILR	C-terminus	0	0	Likely Pathogenic	EIEE

a variant observed in Patients 1 and 2.

Six heterozygous in-frame SCN8A variants, based on transcript NM_014191.4, identified in the ClinVar database (July 2021). VUS, variant of uncertain significance; EIEE, early infantile epileptic encephalopathy; NS, not specified. Classification and clinical information provided by the ClinVar database.

**TABLE 2:** In-frame *SCN8A* variants observed in the gnomAD database.

Transcript change	Protein change	Location	gnomAD v2.1.1allele count	gnomAD v3.1.1 allele count
c.1401_1409delTGAAGAAGG	p.E468_G470del	DI-DII Linker	0	1
c.1615_1617delAAA	p.K539del	DI-DII Linker	1	1
c.1855_1863delGGCTACAGC	p.G619_S621del	DI-DII Linker	35	18
c.1855_1863dupGGCTACAGC	p.G619_S621dup	DI-DII Linker	7	10
c.1952_1972dupTCATCGGCGGCCCCGGCTCCC	p.L651_S657dup^a^	DI-DII Linker	0	1
c.1962_1982dupCCCCGGCTCCCACATCGGCGG	p.P655_G661dup^a^	DI-DII Linker	1	0
c.2025_2027delGAA	p.K676del	DI-DII Linker	2	0
c.2100_2102delAAT	p.I700del	DI-DII Linker	1	0
c.3225_3245dupGAAGTACATCATTGATGAGGA	p.E1075_E1081dup	DII-DIII linker	2	0
c.5235_5237delCTT	p.F1746del	DIVS6	1	0
c.5895_5897delAAG	p.R1966del	Cytoplasmic Tail	1	1

a variant also observed in the ClinVar database.

Eleven heterozygous in-frame SCN8A variants, based on transcript NM_014191.4, were identified in the gnomAD database, v2.1.1 and v3.1.1 (updated July 2021). Clinical data is not available for these individuals.

In summary, it is likely that additional in-frame *SCN8A* variants will be identified as more patients undergo whole exome and genome sequencing, further expanding the genetic landscape of *SCN8A-*associated disease, and potentially posing challenges for genetic counseling and precision therapy. Due to the rare nature of in-frame *SCN8A* variants, it is currently unclear if penetrance is reduced for this class of variants. Loss-of-function *SCN8A* variants (nonsense, frameshift) have been previously reported to exhibit incomplete penetrance (Trudeau et al., 2006); therefore, penetrance may depend on the functional consequence of the individual in-frame variant. The ΔIRL and ΔVIR *Scn8a* mouse lines will provide the opportunity to further study genotype-phenotype relationships in *SCN8A*-related disease and will assist in the identification of appropriate treatments for patients with this class of *SCN8A* mutation.

## Data Availability

The human datasets presented in this article are not readily available because of ethical and privacy restrictions. Requests to access the datasets should be directed to the corresponding author(s).
